# Work-related exposure to violence or threats and risk of mental disorders and symptoms: a systematic review and meta-analysis

**DOI:** 10.5271/sjweh.3877

**Published:** 2020-07-01

**Authors:** Laura A Rudkjoebing, Ane Berger Bungum, Esben Meulengracht Flachs, Nanna Hurwitz Eller, Marianne Borritz, Birgit Aust, Reiner Rugulies, Naja Hulvej Rod, Karin Biering, Jens Peter Bonde

**Affiliations:** Department of Occupational and Environmental Medicine, Bispebjerg University Hospital, Copenhagen, Denmark; National Research Centre for the Working Environment, Copenhagen, Denmark; Department of Public Health, University of Copenhagen, Copenhagen, Denmark; Department of Psychology, University of Copenhagen, Copenhagen, Denmark; Department of Occupational Medicine, Danish Ramazzini Centre, Regional Hospital West Jutland – University Clinic, Herning, Denmark

**Keywords:** Key terms anxiety, burnout, depression, psychological distress, sleep disturbance, workplace violence

## Abstract

**Objective:**

This review aimed to examine systematically the epidemiological evidence linking work-related exposure to violence and threats thereof with risk of mental disorders and mental ill-health symptoms.

**Methods:**

We searched PubMed, EMBASE, PsycINFO and Web of Science to identify original studies that provide quantitative risk estimates. The evidence was weighted according to completeness of reporting, potential common method bias, and bias due to differential selection and drop out, selective reporting, and misclassification of exposure and outcome.

**Results:**

We identified 14 cross-sectional and 10 cohort studies with eligible risk estimates, of which 4 examined depressive disorder and reported an elevated risk among the exposed [pooled relative risk (RR) 1.42, 95% confidence interval (CI) 1.31–1.54, I^2^=0%]. The occurrence of depressive and anxiety symptoms, burnout and psychological distress was examined in 17 studies (pooled RR 2.33, 95% CI 3.17, I^2^=42%), and 3 studies examined risk of sleep disturbance (pooled RR 1.22, 95% CI 1.09–1.37, I^2^=0%). In most studies, common method bias and confounding could not be ruled out with confidence and strong heterogeneity in most outcome definitions invalidate the strict interpretation of most pooled risk estimates.

**Conclusion:**

The reviewed studies consistently indicate associations between workplace violence and mental health problems. However, due to methodological limitations the causal associations (if any) may be stronger or weaker than the ones reported in this study. Prospective studies with independent and validated reporting of exposure and outcome and repeated follow-up with relevant intervals are highly warranted.

Within the context of work, violence and threats thereof has been recognized as a widespread challenge in numerous studies. Estimates of the frequency vary considerable in the literature according to occupational setting, definitions, and measurement methods (1). In a 2018 Danish survey among 39 000 randomly selected employees aged 18–64 years, 5.8% reported exposure to physical workplace violence and 8.4% reported threats of violence at their workplace during the last 12 months (arbejdsmiljodata.nfa.dk). Workplace violence and threats thereof are reported to be highly prevalent in the healthcare sector and among social workers, teachers, police and prison personnel (2–14).

Though violence in the context of work is a recognized problem the definition of workplace violence is unclear. The International Labor Organization provides a broad definition of workplace violence as “Any action, incident or behavior that departures from reasonable conduct in which a person is assaulted, threatened, harmed, injured in the course of, or as a direct result of, his or her work”. Thus violence can be either physical (such as attacks and beating) or psychological (such as threats and harassment). Since workplace violence is a widespread challenge, it is important to gain knowledge about its possible adverse consequences on mental health. At least three reviews have indicated that violence and threats thereof at the workplace are associated with increased risk of mental ill health (1, 15, 16). However, as reviews were descriptive and narrative in design, they neither provided meta-analyses nor systematically assessed risk of bias or grading of the strength of the evidence.

The hypothesis that workplace violence causes mental disorders is supported by the evidence that exposure to very severe psychological trauma of a catastrophic nature may result in severe psychological disorders, ie, posttraumatic stress syndrome (PTSD) during the following months (15). Although work-related violence and threats may be of catastrophic nature they are most often less severe. On the other hand, work-related violence is often prolonged or repeated, which may contribute to increased risk of mental disorders (16).

Thus, the objective of this article is to review systematically, meta-analyze and critically evaluate the epidemiological evidence for causal relations between violence or threats thereof at work and the risk of depressive and anxiety disorders (primary outcomes) and mental ill-health symptoms (secondary outcomes), respectively. In this review, work-related violence and threats thereof were defined as direct physical assault and/or threats of physical assault taking place in the work context. Verbal aggressive or hostile communication/behavior and bullying/harassment that do not include physical assault or threats about physical assault were not included.

## Methods

A review protocol was registered at PROSPERO (Prospero.org, number CRD42018087076) and the review was conducted and reported according to the PRISMA 2009 guidelines (supplementary data, www.sjweh.fi/show_abstract.php?abstract_id=3877, appendix A).

### Search strategy and selection criteria

We searched the databases PubMed, EMBASE, Psyc­INFO and Web of Science (supplementary data, appendix B) and supplemented this by sifting through reference lists in retrieved papers and reviews.

We aimed to identify journal articles providing quantitative risk estimates for mental disorders and caseness of mental health symptoms in relation to physical violence or threats thereof at the workplace with the following inclusion criteria: (i) Fulltext papers in English in journals with peer-review published from the start of the current database up to 1 May 2018; (ii) Exposures: violence at the workplace defined as being exposed to direct aggressive physical assault or to threats of physical violence (oral or written intimidating or threatening statements, threatening behavior such as a raised fist, advancing behavior and stalking). Verbal assault and hostile behavior, bullying and sexual harassment were not included unless they explicitly involved physical violence or threats thereof; (iii) Primary outcomes: mental health disorders (depressive disorder (ICD10 F32–33) and anxiety disorder (ICD10 F40–41), but not PTSD and adjustment disorders (ICD10 F43) since these disorders are defined by their cause and are, therefore, not eligible in controlled observational studies of exposure–outcome relations in which the outcome must be defined independent of the exposure; (iv) Secondary outcomes: depressive symptoms, anxiety symptoms, psychological distress, burnout, comprising symptoms such as being physically or emotionally exhausted and feeling tired including emotional exhaustion and fatigue, and disturbed sleep and; (v) Exposure taking place within one year before execution of the study in order to exclude studies with a long or poorly defined time span between exposure and outcome (17). Only one study addressing life-time exposure to violence was excluded as a result of this criterion (18); (vi) Effect measures: indicators of relative or absolute risk of disorders or symptom caseness. Seven (19–25) cross-sectional or longitudinal studies addressing a diversity of symptom outcomes reporting correlation or regression coefficients based upon continuous exposure and/or outcome scores were not included since these studies were not eligible for meta-analyses based upon relative risk measures. Eight other studies were excluded because they did not provide any measure of association at all (8, 26–32).

### Data extraction

Descriptive information and risk estimates were retrieved from each publication using a standardized form (table 1 and supplementary data, appendix C, tables S1–7). If physical violence was not distinguished from threats of violence, we categorized the outcome as the latter. If the relevant relative risks (RR) were not reported but data were available, we computed risk estimates and confidence intervals (CI) (five studies (33–37).

**Table 1:** Characteristics of studies addressing psychiatric disorders and prescription of antidepressive medicine. [NA=not available; RR=relative risk.]

Author and country	Population	Follow-up	Exposure ascertainment	Outcome	Outcome ascertainment	Outcome prevalence in the reference group	Comparison	RR	95% CI	Report completion (0–8)	Bias score 0–5
Wieclaw et al 2006, Denmark (2)	Patients from the Danish Psychiatric Central Research Register (N=14 166) and matched controls (age, sex and time) from Statistics Denmark’s Integrated Database for Labor Market Research (N=58 060)	12 months	Job exposure matrix	Affective disorders (F30–39)	Register data, hospital records	NA	Threats men (0% as reference):			8	2
							0–20%	1.07	0.96–1.19		
							>20%	1.17	0.92–1.48		
							Threats women (0% as reference):				
							0–20%	1.14	1.04–1.26		
							>20%	1.48	1.23–1.79		
							Violence men (0% as reference):				
							0–14%	1.03	0.90–1.18		
							>14%	1.45	1.27–1.65		
							Violence women (0% as reference):				
							0–14%	1.25	1.03–1.23		
							>14%	1.48	1.18–1.86		
Geiger-Brown et al 2007, USA (48) ^a^	Home care workers Wave 1 (N=1643) Wave 2 (N=1198). Response rate 88%	6 months	Telephone interview, five questions about the level of violence	Depression	Revised Center for Epidemiologic Studies Depression Scale (RCES-D) 20 items	6.6 %	Threats vs none:	3.74	0.82–17.12	8	2
							Violence vs none:	7.29	0.78–68.24		
							Both threats and violence:	10.8	3.87–30.19		
Madsen et al 2011, Denmark (47) ^a^	Random sample of the working-age population in Denmark (N=15 246) Response rate 60–80%	3.5 years	Self-administered questionnaire and interviews, two questions	Anti-depressants	Register of Medicinal Products Statistics	Anti-depressiva 4.1%	Violence yes vs no:	1.38	1.09–1.75	8	1
Dement et al 2014, USA (39) ^a^	Nurses, nurses’ aides, police officers, security workers (N=9884)	6 years	Register, (workers compensation claims, incident reports, and OSHA logs).	Prescriptions for anti-depression or anti-anxiety drugs/ register	National Drug Codes (NDC) contained within the line-item pharmacy claims	Anti-depressiva and anxiolytics 14.8%	Reporting an incident vs not:			8	1
							Male	1.39	0.88–2.21		
							Female	1.51	1.03–2.22		

a Cohort study.

### Quality assessment

Two authors independently reviewed the papers and considered the quality of each study using the instruments listed below. Discrepancies were resolved by consensus or involvement of a third author.

*Completeness of reporting*. Each publication was evaluated for completeness of reporting by considering the following study characteristics modified after Bonzini et al (38): study design, definition of study population, recruitment procedure, response rate, exposure ascertainment, outcome ascertainment, data analyses and statistical modelling.

We evaluated whether each of these study characteristics were described (score=1) or not (score=0). Giving equal weight to each of the eight items, we considered completeness of reporting as sufficient if the sum of the 0/1 scores for each paper was ≥6 (38). Completeness of reporting is not a direct measure of scientific quality or validity, but a measure of reporting quality.

*Bias and confounding*. We identified a priori the following potential types of bias of particular importance in the field: (i) *Selection bias* due to differential participation in cross-sectional and case–control studies or differential drop-out in cohort studies with a risk of overrepresentation of exposed with disease. This may cause bias in either direction; (ii) *Common method bias* resulting from self-report of both exposure and outcome. This applies in particular to cross-sectional and case–control studies but may also affect cohort studies and is expected to inflate risk estimates; (iii) *Non-differential misclassification* between exposure and outcome resulting from crude or inaccurate methods of ascertainment. This is expected to deflate risk estimates; (iv) *Selective reporting of results* in studies with multiple analyses, which is expected to inflate risk estimates.

Confounding was considered unresolved unless sex, age, and socioeconomic status (measured with education, income or occupational class) were accounted for by analysis or design. For each type of bias, the risk was rated as high (score=1) or low (score=0), and we categorized a study at higher risk of bias when the sum of scores was ≥2.

### Meta-analysis

In studies where exposure was divided into levels by severity or frequency and risk estimates were reported according to these levels, the highest level versus the reference category was selected for the meta-analysis. We computed a summary risk estimate across all studies grouped by exposure and by primary and secondary outcomes. If a true risk exists, it is likely to differ across studies. Therefore random effects models were used for weighting odds ratios or equivalent [RR or hazard ratios] by the inverse variance. Heterogeneity was assessed by the I^2^ statistic. Meta-analyses were carried out in R version 3.4.4 using packages meta, metaphor and forest plot.

In supplementary analyses, we excluded studies with potential bias or missing information on two or more of the eight study characteristics that we evaluated. Potential publication bias was visualized by funnel plots displaying risk estimate variance by risk estimate.

## Results

**Figure 1:**
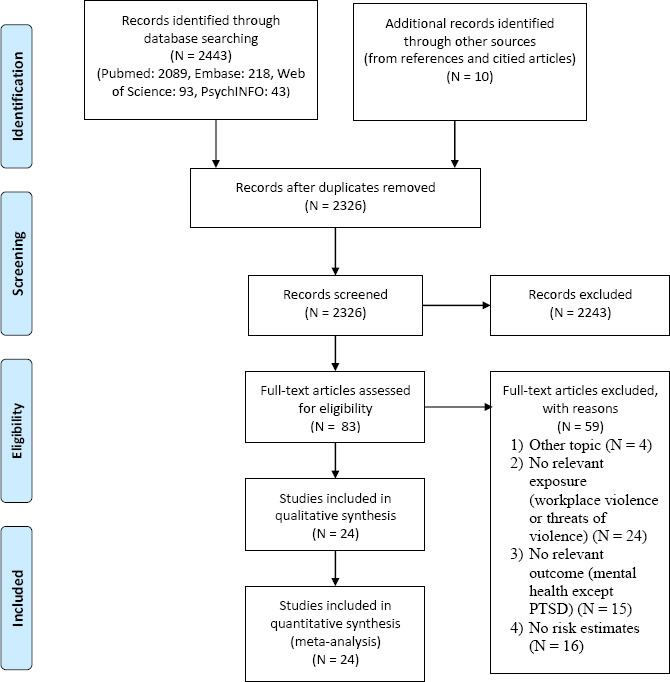
Prisma flow diagram.

We identified 24 independent studies that fulfilled the eligibility criteria (figure 1): 10 cohort or nested case–control studies and 14 cross-sectional studies. Characteristics of the studies stratified by outcome are provided in table 1 (primary outcomes) and the online supplementary data, appendix C, tables S1–6 (secondary outcomes).

Information on exposure to workplace violence or threats thereof was retrieved by self-reports in questionnaires in 16 studies (33–37, 40–42, 44, 45, 47, 49, 50, 53–55), interviews in 6 studies (43, 46, 48, 51, 56, 57), a job exposure matrix (2), and records of compensation claims (39). Questions were most often 1- or 2-item questions such as “Have you been exposed to physical violence at your workplace during the last 12 months?” without further specification. These studies reported a prevalence of threats of violence of 0.8–20% and for violence a prevalence of 0.7–42%.

Two studies (40, 41) specified a list of 13–18 items of different forms of violent incidents and threats (prevalence of threats 18% and violence 23%), and eight studies (33–35, 42–46) applied multi-item scales developed in earlier research such as the Violent Incidence Form (VIF) with reported prevalences of threats of violence and violence of 12–27.7% and 9.2–75%, respectively (33, 42, 43), the Experience of Assault Questionnaire (prevalence of violence of 63.4%) (34) and the Workplace Violence in the Health Sector Country Case Studies Research Instruments reporting a prevalence of threats of violence of 24.2% and of violence from 5–15.6% (35, 44–46). One study used a job exposure matrix to assess the exposure to threats of violence and violence with a prevalence of 5.1–6.9% and 1.1–3.3%, respectively (2).

For outcome occurrence, prevalence also varied substantially across studies. The range of prevalence of depression in the reference group was 4–14%, (see table 1). Prevalence of depressive and anxiety symptoms in the reference group ranged 15–57% and 13–26%, respectively. For burnout, prevalence ranged 3–64%, for psychological distress 17–39%, and for sleep problems 5–30% (supplementary data, appendix C, tables S1–6). Completeness of reporting was satisfactory in most studies, but incomplete in seven, mostly due to a lack of information on data analyses and recruitment procedures.

The 24 studies provided a total of 41 risk estimates (none with absolute measures of risk) on the association between violence/threats of violence and mental health outcomes of which 39 were above unity. The overall summary RR was 1.70 (95% CI 1.47–1.95). Since the difference in the summary risk of exposure to violence (RR 1.47, 95% CI 1.28–1.68) and exposure to threats of violence (RR 1.82, 95% CI 1.43–2.31) was minor, the results are presented together in the following.

### Primary outcomes (psychiatric disorders)

**Figure 2:**
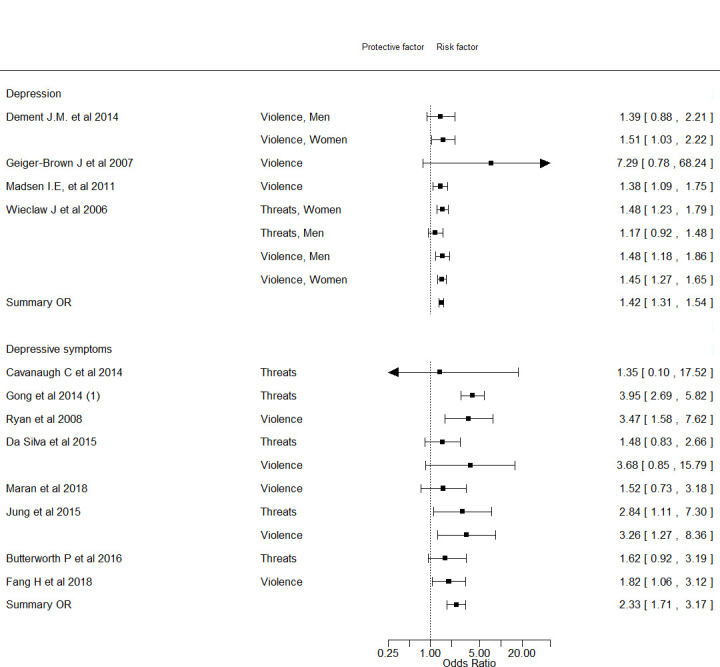
Forest plot on the association between violence/threats of violence and risk of depression and depressive symptoms.

Figure 2 depicts the four studies that addressed risk of depressive disorder (2, 39, 47, 48). The weighted averaged RR according to these studies was 1.42 (95% CI 1.31–1.54, I^2^=0%), Only one, a registry-based study, explicitly addressed risk of medically diagnosed depressive disorder (and other mood disorders), while two cohort studies used prescription of anti-depressive medication as a proxy measure of depressive disorder (39, 47). The last study used the revised version of the 20 item CES-D, possibly providing more reliable data on depressive disorder as evidenced by a prevalence of 6.6% in the reference group (48).

### Secondary outcomes (mental ill-health symptoms and sleep problems)

Figure 2 also shows the estimates for *depressive symptoms* that were addressed in eight studies (ten risk estimates) (33, 34, 37, 45, 46, 49–51), including one cohort study. All studies reported a RR above unity (pooled RR 2.33 (95% CI 1.71–3.17, I^2^=42%)). The prevalence of depressive symptoms in the reference group was 15–57% and is therefore unlikely to indicate major depression (supplementary data, appendix C, table S1). Completeness of reporting score varied between four and eight and all studies were vulnerable to at least one type of bias (supplementary data, appendix C, table S7).

**Figure 3:**
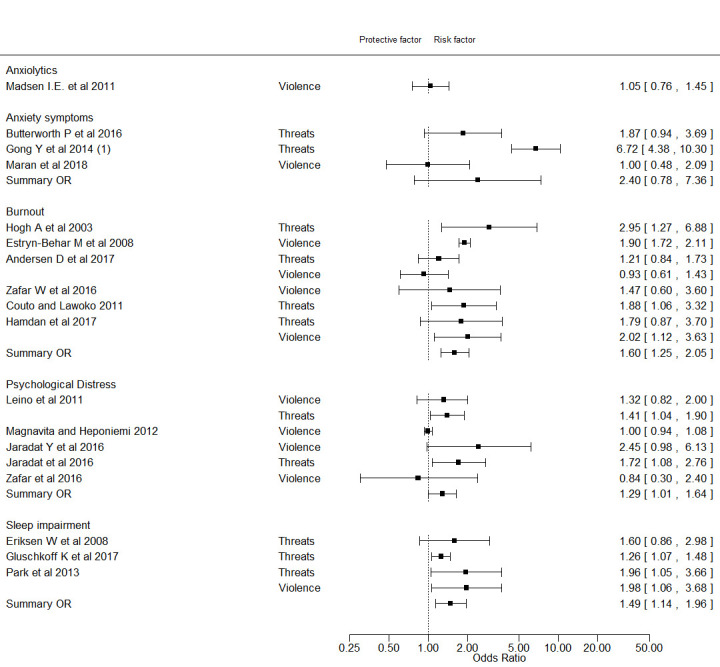
Forest plot on the association between violence/threats of violence and treatment with anxiolytics, anxiety symptoms, burnout, anxiety symptoms and sleep impairment.

Figure 3 depicts estimates for anxiety, anxiety symptoms, psychological distress, burnout and sleep problems. *Anxiety*, assessed by prescribed anxiolytics was examined in one study that did not find an association with exposure to violence (47). *Anxiety symptoms* were reported in three studies (33, 50, 51) with a summary RR of 2.40 (95% CI 0.78–7.36, I^2^=90%). Completeness of reporting score ranged from five to eight and all studies exhibited two or three types of potential bias that we rated as important prior to our study (supplementary data, appendix C, table S7).

*Burnout*, including emotional exhaustion and fatigue, was examined in six studies (supplementary data, appendix C, table S5) (41, 43, 44, 52–54): three cohort studies and three cross-sectional studies. The summary estimate across all six studies was 1.60 (95% CI 1.25–2.05, I^2^=57%), figure 3. Completeness of reporting score ranged from six to eight. In all studies we assessed potential bias (supplementary data, appendix C, table S7).

*Psychological distress* was measured in four studies (35, 40, 42, 44) (six risk estimates) with four risk estimates above unity (supplementary data, appendix C, table S4). The summary estimate across all studies was 1.29 (95% CI 1.01–1.64, I^2^=58%), figure 3. Completeness of reporting score ranged from five to seven. In all studies, we assessed likely bias (supplementary data, appendix C, table S7).

*Sleep problems* were addressed in two cohort studies (36, 55) and one cross-sectional study (56) (four risk estimates). The summary risk estimate across all studies was 1.49 (95% CI 1.14–1.96, I^2^=0%) and the corresponding risk estimate for the two cohort studies were 1.22 (95% CI 1.09–1.37, I^2^ = 0%). Completeness of reporting score seven or eight. Risk of bias was considered unlikely in only one study (supplementary data, appendix C, table S7).

### Exposure–response

Seven studies examined the exposure–response relation according to level or frequency of violent acts and five of these studies found the risk to be increased in parallel with increasing frequency of exposure (2, 43, 46, 54, 57), including one of the studies on depressive disorder (2). In two studies, the findings were inconsistent (36, 40).

### Study design

Considering both violence and threats thereof and all outcomes together, the pooled estimates for the 10 cohort and case–control studies (RR 1.36, 95% CI 1.17–1.58) tended to be lower than the pooled estimates for the 14 cross-sectional studies (RR 1.92, 95% CI 1.55–2.37).

### Publication bias

**Figure 4:**
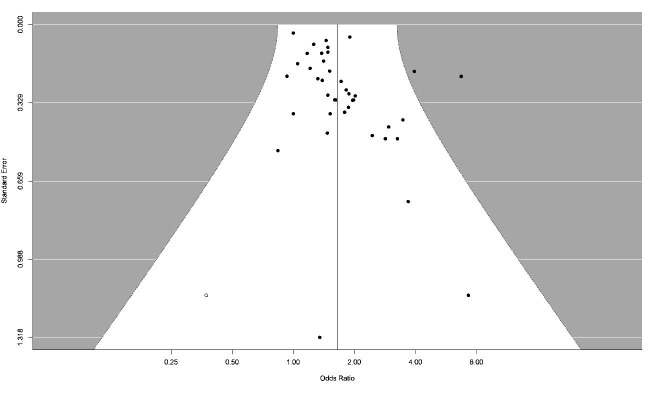
Funnel plot of all included studies.

A funnel plot demonstrating the relationship between precision and magnitude of the risk estimate provides no strong indication that larger or more precise studies systematically report risks of smaller magnitude than small studies (figure 4). Thus, publication bias is unlikely.

## Discussion

In this systematic review of the epidemiological evidence on the relation between workplace-related violence and threats thereof and mental health problems, we included 14 cross-sectional and 10 cohort studies.

The criteria for exclusion (verbal assault and hostile behavior, bullying, sexual assault and harassment) can be difficult to distinguish from the criteria for inclusion (threats of violence). The data on exposure depends on the perception, appraisal, and the state of the victim of the verbal assault, hostile behaviors or threats, making this an issue for comparability of studies. Threats were defined as verbal threats of directly physical violence or threats such as raised fits and advancing behavior. The strict definition of threats of violence applied in this review has reduced the number of eligible studies, but not necessarily the number of high-quality studies and studies addressing medical mental health outcomes and, therefore, may have few – if any – implications for the conclusions at which we arrive. Exposure to violence is easier to define and distinguish from the other mentioned behaviors though sexual harassment (which we excluded in this review) in some situations can be perceived as violence.

Physical violence (bodily attacks) may be assumed in general to represent more severe exposure than threats of violence, and the pooled risk estimates we found for threats of violence and violence respectively also showed this tendency. However, the difference was small and the CI overlapped. Therefore, we argue that the most parsimonious approach is to study violence and threats thereof together.

The validity of the instruments used to measure exposure is another issue of concern. Half of the studies (12 studies) used 1–3 single questions, which may cause differential misclassification. However, the prevalences of violence/threats found in these studies (0.7–42%) are not much different compared to those found with the more validated measurement instruments (5–75%). The study using a job exposure matrix reported prevalence of threats of violence of 5.1–6.9% and 1.1–3.3% for violence, which are a bit lower than those found by the self-reported data. Since no systematic difference is observed across the measurements methods, analyses stratified by type of instrument is hardly informative. However, the very broad range of exposure prevalence across all the included studies reflects the strong heterogeneity, problems related to comparability, and emphasizes the lack of a uniform definition of workplace violence.

Fifteen excluded studies were not informative with respect to the primary outcome of this review (mental health disorders) because no risk estimates were provided. Although seven of these studies did provide alternative measures of association, they were not eligible for meta-analyses based on RR estimates of caseness and were therefore not considered further.

Consistency of risk estimates across studies with different designs, settings, and geographical regions was remarkable with almost all studies reporting an elevated risk in relation to work-related violence and threats thereof. In relation to the triangulation theory (58), this consistency across different study designs and populations supports a causal association. However, this consistency needs to be viewed in the light of the high variation in baseline outcome prevalence in the reference groups, which for instance for depressive symptoms ranged from 15–57%. Most likely this variation is due to differences in outcome definition and ascertainment rather than being a reflection of large variation of the occurrence of outcomes. None of the studies explicitly ascertained the depressive disorder diagnosis by a psychiatric interview, which is regarded as the most reliable method (59, 60). Moreover, the variety in the methods for assessment of violence and the quality of these measurements make it difficult to find clear causal relations.

### Bias causing inflation of the risk estimates.

Information on exposure as well as outcome was obtained by questionnaire or interview in 20 of the 24 studies and are therefore not mutually independent observations. Since reduced psychological well-being or even a predisposition for mood disorders may influence perception and reporting of violence or threats, there is a risk for so-called common method bias, which is expected to inflate risk estimates (61–63) although the opposite may also be true (66). It might be speculated that reporting of threats is more prone to bias than reporting of violence which is more easy to prove – at least when it comes to severe cases of physical assault. However, the summary risk estimates of the two categories of exposure do not strongly deviate. We also assessed biased results due to selective reporting. Since we retrieved risk estimates by predefined criteria without considering the objectives of the included papers, bias due to selective reporting is unlikely.

### Bias causing deflation of the risk estimates

Selective inclusion where individuals who are healthy at baseline may represent a more robust survivor population – either because employees with mental health problems avoid jobs with a high potential for violence or because employees who became victim to violence and subsequently encountered mental health problems may have left the job before entering the study. In addition, some people would never consider working in a psychiatric ward or a prison, so self-selection into job might also play a role. Moreover, if violence is triggering a disorder without delay – as would be expected – and victims recover within some months, it may be difficult to detect an increased risk in follow-up studies with a long time-span from baseline reporting of violence and ascertainment of the outcome at follow-up. This could be the case for the cohort studies included in this review where follow-up intervals in most studies were two years. As shown, the summary estimates in the cohort and case–control studies tended to be lower than for the cross-sectional studies.

Another source of bias that likely results in attenuated risk estimates is misclassification of exposure in studies using job exposure matrices, but this only applied to one study in this review.

### Confounding

We evaluated confounding by sex, age, and socioeconomic status according to the a priori published protocol. In addition, mental health status may profoundly influence reporting of being subjected to violence or threats thereof, aggressive behaviors of clients or patients and risk of later mental disorders or distress. Evidence for an association of mental health status and risk of bullying was found in a prospective study showing that individuals reporting mental distress exhibited a higher risk of being bullied two years later (64), although another explanation for this result could also be that individuals exhibiting signs of mental distress were more often targeted by perpetrators of bullying. However, this may not be a major source of bias in cohort studies included in this review since baseline mental health was controlled for in all the cohort studies except two (41, 54).

### Concluding remarks

The reviewed studies consistently indicated associations between workplace violence and mental health problems. However, due to the methodological limitations of most of the studies, bias and confounding could not be ruled out with confidence and causal associations between violence/threats of violence and mental disorders and mental health symptoms (if any) may be stronger or weaker than the pooled estimates from the meta-analyses. Prospective studies with independent and validated reporting of exposure and outcome and repeated follow-up with relevant intervals are highly warranted.

## Supplementary material

Supplementary material
