# Supplementary material for: Work-related exposure to violence or threats and risk of mental disorders and symptoms: a systematic review and meta-analysis
Source: Scand J Work Environ Health. 2020 Jul 1;46(4):339–49. doi: 10.5271/sjweh.3877 (PMC8506313; doi:10.5271/sjweh.3877)
Supplement: Supplementary material [file SJWEH-46-339-S001.pdf]

Work-related exposure to violence or threats and risk of mental disorders and symptoms: a systematic review and meta-analysis [1]

by Laura A Rudkjoebing, MD,[2] Ane Berger Bungum, BSc, Esben Meulengracht Flachs, PhD, Nanna Hurwitz Eller, DMSc, Marianne Borritz, MD, PhD, Birgit Aust, MSc, PhD, Reiner Rugulies, MPH, PhD, Naja Hulvej Rod, MS, PhD, Karin Biering, PhD, Jens Peter Bonde, MD, PhD

1. Supplementary material
2. Correspondence to: Laura Aviaja Rudkjoebing, Department of Occupational and Environmental Medicine, Bispebjerg University Hospital, Bispebjerg Bakke 23, DK-2400 Copenhagen NV, Denmark. [E-mail: Laura.aviaja.rudkjoebing.01@regionh.dk]

Appendix A. PRISMA 2009 checklist

Section/topic	#	Checklist item	Reported on page #
**TITLE**			
Title	1	Identify the report as a systematic review, meta-analysis, or both.	1
**ABSTRACT**			
Structured summary	2	Provide a structured summary including, as applicable: background; objectives; data sources; study eligibility criteria, participants, and interventions; study appraisal and synthesis methods; results; limitations; conclusions and implications of key findings; systematic review registration number.	3
**INTRODUCTION**			
Rationale	3	Describe the rationale for the review in the context of what is already known.	4
Objectives	4	Provide an explicit statement of questions being addressed with reference to participants, interventions, comparisons, outcomes, and study design (PICOS).	4
**METHODS**			
Protocol and registration	5	Indicate if a review protocol exists, if and where it can be accessed (e.g., Web address), and, if available, provide registration information including registration number.	5
Eligibility criteria	6	Specify study characteristics (e.g., PICOS, length of follow-up) and report characteristics (e.g., years considered, language, publication status) used as criteria for eligibility, giving rationale.	5-6
Information sources	7	Describe all information sources (e.g., databases with dates of coverage, contact with study authors to identify additional studies) in the search and date last searched.	5
Search	8	Present full electronic search strategy for at least one database, including any limits used, such that it could be repeated.	Appendix B
Study selection	9	State the process for selecting studies (i.e., screening, eligibility, included in systematic review, and, if applicable, included in the meta-analysis).	6
Data collection process	10	Describe method of data extraction from reports (e.g., piloted forms, independently, in duplicate) and any processes for obtaining and confirming data from investigators.	6
Data items	11	List and define all variables for which data were sought (e.g., PICOS, funding sources) and any assumptions and simplifications made.	6, Appendix C
Risk of bias in individual studies	12	Describe methods used for assessing risk of bias of individual studies (including specification of whether this was done at the study or outcome level), and how this information is to be used in any data synthesis.	7
Summary measures	13	State the principal summary measures (e.g., risk ratio, difference in means).	6
Synthesis of results	14	Describe the methods of handling data and combining results of studies, if done, including measures of consistency (e.g., I^2) for each meta-analysis.	8
Risk of bias across studies	15	Specify any assessment of risk of bias that may affect the cumulative evidence (e.g., publication bias, selective reporting within studies).	8
Additional analyses	16	Describe methods of additional analyses (e.g., sensitivity or subgroup analyses, meta-regression), if done, indicating which were pre-specified.	8
**RESULTS**			
Study selection	17	Give numbers of studies screened, assessed for eligibility, and included in the review, with reasons for exclusions at each stage, ideally with a flow diagram.	Figure 1
Study characteristics	18	For each study, present characteristics for which data were extracted (e.g., study size, PICOS, follow-up period) and provide the citations.	Appendix C
Risk of bias within studies	19	Present data on risk of bias of each study and, if available, any outcome level assessment (see item 12).	Appendix C, Table 7
Results of individual studies	20	For all outcomes considered (benefits or harms), present, for each study: (a) simple summary data for each intervention group (b) effect estimates and confidence intervals, ideally with a forest plot.	Fig. 2-3
Synthesis of results	21	Present results of each meta-analysis done, including confidence intervals and measures of consistency.	Fig. 2-3, 9-12
Risk of bias across studies	22	Present results of any assessment of risk of bias across studies (see Item 15).	9-12
Additional analysis	23	Give results of additional analyses, if done (e.g., sensitivity or subgroup analyses, meta-regression [see Item 16]).	11-12
**DISCUSSION**			
Summary of evidence	24	Summarize the main findings including the strength of evidence for each main outcome; consider their relevance to key groups (e.g., healthcare providers, users, and policy makers).	12-14
Limitations	25	Discuss limitations at study and outcome level (e.g., risk of bias), and at review-level (e.g., incomplete retrieval of identified research, reporting bias).	13-14
Conclusions	26	Provide a general interpretation of the results in the context of other evidence, and implications for future research.	14
**FUNDING**			
Funding	27	Describe sources of funding for the systematic review and other support (e.g., supply of data); role of funders for the systematic review.	15

Appendix B. Systematic literature search specification
Work-related exposure to violence or threats of violence and risk of mental disorders: a systematic review and meta-analysis.
(alternative exposures MeSH/TIAB AND alternative outcomes (MeSH/TIAB) AND alternative designs (TIAB)
(inclusion criteria: original peer reviewed full text papers in English and human studies)

Exposure	Outcome	Design
workplace violence (MesH),<br>threats,<br>assault,<br>aggression,<br>battery,<br>pushing, hitting with an object, hitting with a body part, slapping, kicking, punching, pinching, scratching, biting, pulling hair, throwing an object, spitting, beating, shooting, stabbing, squeezing, twisting, rape<br>shaking fists, throwing furniture, destroying property	mental disorder (MeSH),<br>depression,<br>depressive symptoms,<br>anxiety,<br>adjustment disorder<br>psychological distress,<br>burnout,<br>sleep,<br>psychotropic drugs,<br>sedativa,<br>hypnotics	cross-sectional,<br>case-control,<br>case-referent,<br>cohort,<br>follow-up,<br>longitudinal,<br>prospective<br>health effects,<br>health outcomes

A PubMed search 16.4.2018 using these search strings results in 2,077 hits.
In the screening process, studies can be excluded in terms of the following reasons: missing relevant exposure, missing relevant outcome, missing a risk estimate or other reasons (which will be specified for each excluded study).

APPENDIX C. Summary of results, Table 1-7

Table 1. Characteristics of studies addressing depressive symptoms. Shaded are cohort studies

**Author Country**	**Population**	**Follow-up**	**Exposure ascertainment**	**Outcome**	**Outcome ascertainment**	**Outcome prevalence in the reference group**	**Comparison**	**RR**	**95% CI**	**Completeness of reporting 0-8**	**Bias score 0-5**
Ryan et al. 2008, USA (34)	Employees at a pediatric state psychiatric hospital N=93	-	Self-administered questionnaire, The Experience of Assault Questionnaire, 23 items	Depressive symptoms	The Beck Depression Inventory-II (BDI-II), 21 items	n.a.	Assaulted (A) vs non-assaulted (NA)	3.47	1.58-7.62	4	3
Cavanaugh et al 2014, USA (37)	Female nurses and nursing personnel N=1044 Response rate 81%	Sixmonths	Self-administered questionnaire, one question	Depressive symptoms	The Center for Epidemiologic Studies depression scale (CESD-10)	20.0 %	Threats or physical workplace violence at baseline yes/no	1.35	0.10-17.52	5	3
Gong et al. 2014, China (50)	Physicians working in public hospitals N=2641 Response rate 96.46%	-	Self-administered questionnaire, one question	Depressive symptoms	The Zung Self-Rating Depression Scale (SDS)	28.1 %	Frequency of conflict and violence - often compared to none (ref)	3.95	2.69-5.82	7	2
Da Silva et al. 2015, Brazil (46)	Physicians, nurses, nursing assistants and community health workers<br><br>N=2940	-	Face-to-face interview, the questionnaire of the WHO multi-country study on women's health and domestic violence	Depressive symptoms	Patient Health Questionnaire, Nine items (PHQ-9)	Depressive symptoms 36.3%<br><br>Probable major depression 16%	Threats vs no threats<br>One/few times<br>Several times<br><br>Physical aggression vs none<br>One/few times<br>Several times	 <br>1.28<br><br>1.48	 <br>0.95-1.74<br><br>0.83-2.66	8	1

	Response rate 93%							1.67<br>3.68	0.91-3.04<br>0.85-15.79		
Jung et al. 2015, Korea (49)	Substitute drivers, N=161	-	Self-administered questionnaire, two questions	Depressive symptoms	The Center for Epidemiologic Studies depression scale (CESD-10)	16.8 %	Verbal violence >4 times a year vs <4 times a year (or none)<br><br>Experienced vs Never experienced physical violence over the past year	2.84<br><br>3.26	1.11-.30<br><br>1.27-8.36	6	2
Butterworth et al. 2016, Australia (51)	Randomly selected residents of Canberra and Queanbeyan (NSW) aged 52-58 years N=1466 Response rate 80%	-	Face-to-face interview and online questionnaire, three single questions	Depressive symptoms	Patient Health Questionnaire Depression Scale (PHQ)	14.6 %	Threats of violence vs no threats of violence	1.62	0.92, 3.19	8	2
Fang et al. 2018, China (45)	Otorhinolaryngologists and nurses N=652 Response rate 83.6 %	-	Self-administered questionnaire, modified version of WHO 2003	Depressive symptoms	Zung self-rating depression scale (SDS)	57.2 %	Physical violence yes/no	1.82	1.06-3.12	7	2
Maran et al. 2018, Italy (33)	Hospital staff in cardiology and oncology N=99	-	Self-administered questionnaire, Violent Incident Form (VIF)	Depressive symptoms	Beck Depression Inventory (BDI)	n.a.	Depression suffering yes/no	1.52	0.73-3.18	5	3

Table 2. Characteristics of the study addressing the prescription of anxiolytic medicine. Shaded rows are cohort studies.

**Author Country**	**Population**	**Follow-up**	**Exposure ascertainment**	**Outcome**	**Outcome ascertainment**	**Outcome prevalence in the reference group**	**Comparison**	**RR**	**95% CI**	**Completeness of reporting 0-8**	**Bias score 0-5**
Madsen et al. 2011, Denmark (47)	Random sample of the working-age population in Denmark N=15,246 Response rate 60-80%	Three and a half year	Self-administered questionnaire and interviews, two questions	Anxiolytics	Register of Medicinal Products Statistics	2.7 %	Violence yes vs no	1.05	0.76-1.45	8	1

Table 3. Characteristics of studies addressing anxiety symptoms. Shaded are cohort studies.

**Author Country**	**Population**	**Follow-up**	**Exposure ascertainment**	**Outcome**	**Outcome ascertainment**	**Outcome prevalence in the reference group**	**Comparison**	**RR**	**95% CI**	**Completeness of reporting 0-8**	**Bias score 0-5**
Gong et al. 2014, China (50)	Physicians working in public hospitals N=2641 Response rate 96.46%	-	Self-administered questionnaire, one question	Anxiety symptoms	The Zung Self-rating Anxiety Scale (SAS)	25.7 %	Frequency of conflict and violence - often compared to none (ref)	6.72	4.38-10.30	7	2

Butterworth et al. 2016, Australia (51)	Randomly selected residents of Canberra and Queanbeyan (NSW) aged 52-58 years N=1466 Response rate 80%	-	Face-to-face interview and online questionnaire, three questions	Anxiety symptoms	Face-to-face interview and online questionnaire, Goldberg Anxiety Scale (nine items)	13.2 %	Threats of violence vs no threats of violence	1.87	0.94- 3.69	8	2
Maran et al. 2018, Italy (33)	Hospital staff in cardiology and oncology N=99	-	Self-administered questionnaire, Violent Incident Form (VIF)	Anxiety symptoms	State-Trait Anxiety Inventory (STAI Y)	n.a.	State anxiety, suffering yes/no	1.00	0.48-2.09	5	3

Table 4. Characteristics of cross-sectional studies addressing psychological distress.

**Author Country**	**Population**	**Follow-up**	**Exposure ascertainment**	**Outcome**	**Outcome ascertainment**	**Outcome prevalence in the reference group**	**Comparison**	**RR**	**(95% CI)**	**Completeness of reporting 0-8**	**Bias score 0-5**
Leino et al. 2011, Finland (40)	Police officers and security guards N=1993<br><br>Response rate 58 %	-	Self-administered questionnaire, one question and a list of 13 items of differed forms of physical violence	Psychological distress	General Health questionnaire (GHQ12)	17%	Physically violent acts none vs<br>Seldom<br>Often<br>very often<br><br>Threats or None vs at least once	 <br><br>1.30<br>1.23<br>1.32<br><br><br>1.41	 <br><br>0.88-1.92<br>0.82-1.82<br>0.87-2.00<br><br><br>1.04-1.90	7	2

Magnavita and Heponiemi. 2012, Italy (42)	Health care workers N=1455<br><br>Response rate 80,1%	-	Self-administered questionnaire, the Violent Incident Form (VIF)	Psychologi-cal 'problems'	General Health questionnaire (GHQ 12)	n.a.	Physical violence vs none	1.00	0.94-1.08	5	3
Jaradat et al. 2016, Palestine (35)	Nurses N=343 Response rate 92.2%	-	Self-administered questionnaire, WHO 2003	Psychological distress	General Health Questionnaire GHQ 30	n.a.	Exposed vs unexposed Violence Threats	2.45<br>1.72	0.98-6.13<br>1.08-2.76	7	2
Zafar et al. 2016, Pakistan (44)	Physicians working in four large hospitals N=179<br><br>Response rate 92.2 %	-	Self-administered questionnaire, WHO 2003	Mental distress (anxiety, depression)	General Health Questionnaire (GHQ12)	39.3%	Physical attack vs no attacks	0.84	0.3-2.4	7	2

Table 5. Characteristics of studies addressing burnout, emotional exhaustion and fatigue. Shaded rows are cohort studies

**Author Country**	**Population**	**Follow-up**	**Exposure ascertainment**	**Outcome**	**Outcome ascertainment**	**Outcome prevalence in the reference group**	**Comparison**	**RR**	**95% CI**	**Completeness of reporting 0-8**	**Bias score 0-5**
Hogh et al. 2003, Denmark (57)	Random sample of Danish citizens/employees N=4961 Response rate 90 %	Five years	Telephone interview, one question	Fatigue	Telephone interview, SF-36 questionnaire, four questions	9.4%	Exposure to violence no or only slightly vs Not much Moderately Very much	1.13<br>1.75<br>2.95	0.73-1.74<br>1.03-2.97<br>1.27-6.88	8	2
Estryn-Behar et al. 2008, Eight European countries	Nurses N=39.898 (NEXT study) Response rate 51 %	One year	Self-administered questionnaire, one question	Burnout	The Copenhagen Burnout Inventory, six items	n.a.	Violence seldom vs Monthly Weekly+	1.38<br>1.90	1.26-1.52<br>1.72-2.11	5	3

(54)											
Couto and Lawoko. 2011, Mozambique (43)	Drivers and conductors working with road passenger transport N=504 Response rate 100%	-	Telephone interviews, the Violent Incident Form (VIF)	Burnout	Maslach Burnout Inventory, General Survey	Mild 30.1% Severe 3.6%	Workplace violence no vs Yes, once or twice Yes several times	0.96<br>1.88	0.57-1.63<br>1.06-3.32	8	1
Zafar et al. 2016, Pakistan (44)	Physicians working in four large hospitals N=179<br><br>Response rate 92.2 %	-	Self-administered questionnaire, WHO 2003	Emotional exhaustion	Maslach Burnout Inventory, Emotional exhaustion, nine items	42.4%	Physical attack vs no attacks	1.47	0.6-3.6	7	2
Andersen et al. 2017, Denmark (41)	Prison personnel N=1741,<br><br>Response rate 61%	One year	Self-administered questionnaire, one question and a checklist of 11 violent incidents and seven different threats of violence.	Burnout	Copenhagen Psychosocial Questionnaire	n.a.	Most exposed quartile vs least exposed three quartiles Violence Threats	0.93<br>1.21	0.61-1.43<br>0.84-1.73	7	4
Hamdan and Hamra. 2017, Palestine (53)	Workers in emergency departments N=444 Response rate 74.5 %	-	Self-administered questionnaire, one question	Burnout	Maslach Burnout Inventory, Human Services Survey	64.8%	Workplace violence yes vs no Violence Threats	2.02<br>1.79	1.12-3.63<br>0.87-3.70	6	2

Table 6. Characteristics of studies addressing disturbed sleep. Shaded are cohort studies

Author Country	Population	Follow-up	Exposure ascertainment	Outcome	Outcome ascertainment	Outcome prevalence in the reference group	Comparison	RR	95% CI	Completeness of reporting 0-8	Bias score 0-5
Eriksen et al. 2008, Norway (36)	Random sample of nurses aids N=4774 Response rate 62 %	Three months	Self-administered questionnaire, one question	Poor sleep	Basic Nordic Sleep questionnaire, one item	29.7 %	Never or very seldom vs<br>Rather seldom<br>Sometimes<br>Rather often<br>Very often or always	 0.87<br>1.08<br>1.77<br>1.60	 0.68-1.13<br>0.86-1.37<br>1.27-2.46<br>0.86-2.98	8	1
Park et al. 2013, Korea (56)	Representative sample of actively working population age 18-65 N=10,039	-	Face to face interviews, two questions	Sleep problems	One question yes/no	5.1 %	Violence no/yes<br>threats no/yes	1.98<br>1.96	1.06-3.68<br>1.05-3.66	7	2
Gluschkoff et al. 2017, Finland (55)	Primary and secondary school teachers N=4988 Response rate 80 %	Two years	Self-administered questionnaire, one question	Sleep disruption	Jenkins sleep problems scale, four items	n.a.	Two years after vs before event	1,26	1.07-1.48	8	0

Table 7. Completeness of reporting and assessment of bias and confounding

Study	Completeness of reporting									Assessment of bias and confounding					
	Study design	Definition of study population	Recruitment procedure	Response rate	Exposure ascertainment	Outcome ascertainment	Data analyses	Statistical modelling	**Sum score (0-8)**	Selection bias	Common method bias	Non-differential misclassification	Selective reporting of results	Confounding	**Sum score (0-5)**
**Studies addressing psychiatric disease**															
Wieclaw et al. 2006	+	+	+	+	+	+	+	+	**8**	0	0	+	0	+	**2**
Geiger-Brown et al. 2007	+	+	+	+	+	+	0	0	**8**	0	+	0	0	+	**2**
Madsen et al. 2011	+	+	+	+	+	+	+	+	**8**	+	0	0	0	0	**1**
Dement et al. 2014	+	+	+	+	+	+	+	+	**8**	0	0	+	0	0	**1**
**Studies addressing depressive symptoms**															
Ryan et al. 2008	0	+	0	0	+	+	0	+	**4**	+	+	0	0	+	**3**
Cavanaugh et al. 2014	+	+	0	+	+	+	0	0	**5**	+	+	0	0	+	**3**
Da Silva et al. 2015	+	+	+	+	+	+	+	+	**8**	0	+	0	0	0	**1**
Jung et al. 2015	0	+	+	0	+	+	+	+	**6**	+	+	0	0	0	**2**
Butterworth et al. 2016	+	+	+	+	+	+	+	+	**8**	0	+	0	+	0	**2**
Fang et al. 2018	+	+	+	+	+	+	0	+	**7**	0	+	0	0	+	**2**
Maran et al. 2018	0	+	+	0	+	+	0	+	**5**	+	+	0	0	+	**3**
**The table continues next page**															
**Study**	**Completeness of reporting**									**Assessment of bias and confounding**					

	Study design	Definition of study population	Recruitment procedure	Response rate	Exposure ascertainment	Outcome ascertainment	Data analyses	Statistical modelling	**Sum score (0-8)**	Selection bias	Common method bias	Non-diffe-rential misclassification	Selective reporting of results	Confoun-ding	**Sum score (0-5)**
**Studies addressing anxiety diagnosis**															
Madsen et al. 2011	+	+	+	+	+	+	+	+	**8**	+	0	0	0	0	**1**
**Studies addressing anxiety symptoms**															
Ryan et al. 2008	0	+	0	0	+	+	0	+	**4**	+	+	0	0	+	**3**
Gong et al. 2014	+	+	+	+	+	+	0	+	**7**	0	+	0	0	+	**2**
Butterworth et al. 2016	+	+	+	+	+	+	+	+	**8**	0	+	0	+	0	**2**
Maran et al. 2018	0	+	+	0	+	+	0	+	**5**	+	+	0	0	+	**3**
**Studies addressing psychological distress**															
Leino et al. 2011	0	+	+	+	+	+	+	+	**7**	+	+	0	0	0	**2**
Magnavita N. 2012	+	+	+	+	0	0	0	+	**5**	0	+	0	+	+	**3**
Jaradat et al. 2016	+	+	0	+	+	+	+	+	**7**	0	+	0	0	+	**2**
Zafar et al. 2016	+	+	+	+	+	+	0	+	**7**	0	+	0	0	+	**2**
**Studies addressing burnout, emotional exhaustion and fatigue**															
Hogh et al. 2003	+	+	+	+	+	+	+	+	**8**	0	+	0	0	0	**1**
Estryn-Behar et al. 2008	+	+	+	+	+	0	0	+	**5**	+	+	0	0	+	**3**
Couto and Lawoko. 2011	+	+	+	+	+	+	+	+	**8**	0	+	0	0	0	**1**
Zafar et al. 2016	+	+	+	+	+	+	0	+	**7**	0	+	0	0	+	**2**
Andersen et al. 2017	+	+	+	+	+	+	0	+	**7**	+	+	0	0	+	**3**

Hamdan and Hamra. 2017	+	+	0	+	+	+	0	+	**6**	0	+	0	0	+	**2**
**Studies addressing disturbed sleep**															
Eriksen et al. 2008	+	+	+	+	+	+	+	+	**8**	0	+	0	0	0	**1**
Park et al. 2013	+	+	+	0	+	+	+	+	**7**	+	+	0	0	0	**2**
Gluschkoff et al. 2017	+	+	+	+	+	+	+	+	**8**	0	0	0	0	0	**0**

Summary of overall results

The studies covered 15 countries, almost one third were from Scandinavia but all continents were represented. Sample sizes varied from small (< 300 workers) to very large in studies with national coverage in specific occupational groups, the median sample size being 6,867, and the total number of participants was around 165,000. Participation rates at baseline were above 80% in nine out of 24 studies and participation rates at follow-up were above 80% in three out of the ten cohort studies. The most frequent occupational groups studied were nurses and other health care professionals (14 studies. Risk estimates were mainly based upon comparisons of respondents reporting exposure to violence or threats of violence versus respondents reporting no such exposure.

Exposure and outcome ascertainment

Information on exposure to workplace violence or threats of violence was retrieved by self-reports in questionnaires in 16 studies, interviews (six studies), a job exposure matrix (one study) (2) and records of compensation claims (one study) (39). Questions were most often one- or two-item questions such as “Have you been exposed to physical violence at your workplace during the last 12 months?” without further specification.

However, two studies specified a list of 13-18 items of different forms of violent incidents and threats(40, 41), and eight studies applied multi-item scales developed in earlier research such as the Violent Incidence Form (VIF) (33, 42, 43), The Experience of Assault Questionnaire (34) or the Workplace Violence in the Health Sector Country Case Studies Research Instruments (35, 44-46). The majority of the studies had the most recent 12 months time period as exposure window but three studies asked about the previous six months(34, 48, 51) and in two studies the time frame was not clearly defined in the questionnaire(54, 57). Data on frequency of exposure the preceding 12 months were obtained in some studies while measures of severity and temporality were scarce. The prevalence of reported exposure varied substantially across studies – from 2.3%(46) to 63.4%(34) for violence and from 0.8%(56) to 75%(43) for threats of violence.

Outcome ascertainment was based upon questionnaire replies (15 studies) or telephone/face-to-face interview (six studies) using different versions of symptom scales such as CES-D (Center for Epidemiologic Studies Depression Scale), BDI (Beck Depression Inventory), GHQ (general health questionnaire), SCL (symptom check list) and the SF-36 vitality scale. Two studies used prescription of anti-depressive pharmaceuticals and one study hospital records to identify cases with depressive disorder (2, 39, 47). Outcome occurrence varied substantially – for instance the prevalence of depressive disorders and depressive symptoms spanned from 4% (47) to 57%(45).
